# Phase angle as an early-warning indicator of glycaemic control in adults with type 1 diabetes mellitus: a cross-sectional study

**DOI:** 10.3389/fendo.2026.1793935

**Published:** 2026-02-26

**Authors:** Lun Zhang, Jiani Zhu, Fan Yang, Tongfen Cui, Xiaoyun Xi, Luying Yang, Ji-Gan Wang, Yunying Cai

**Affiliations:** 1 Department of Clinical Nutrition, The First People’s Hospital of Yunnan Province, The Affiliated Hospital of Kunming University of Science and Technology, Kunming, Yunnan, China; 2 Department of Internal Medicine, Buga Town Health Center, Zhaotong, Yunnan, China; 3 Department of Endocrine Metabolism, Changning County People’s Hospital, Baoshan, Yunnan, China; 4 Department of Clinical Nutrition, Baoshan Second People’s Hospital, Baoshan, Yunnan, China; 5 Department of Pediatrics, Maternal and Child Health Hospital of Guangxi Zhuang Autonomous Region, Nanning, China; 6 Department of Endocrine Metabolism, The First People’s Hospital of Yunnan Province, The Affiliated Hospital of Kunming University of Science and Technology, Kunming, Yunnan, China

**Keywords:** adult, correlation, glycated hemoglobin, phase angle, type 1 diabetes

## Abstract

**Background:**

For patients with poor glycemic control of adult type 1 diabetes mellitus (T1DM), in addition to managing blood glucose levels, it is worth exploring further as an early warning indicator of blood glucose control.

**Methods:**

56 adults with T1DM, aged between 18 and 70 years, were included in the study. Data on body composition and laboratory indicators, including phase angle values and HbA1c levels, were collected. Statistical analysis was conducted to determine the correlation and the strength of association between the phase angle, and HbA1c levels.

**Results:**

Pearson’s correlation and linear regression models indicated a negative correlation between phase angle and HbA1c levels, even after controlling for age and weight in both males and females.

**Conclusion:**

The results indicate a significant negative relationship between Phase angle and HbA1c levels. Beyond being a simple body composition parameter, Phase angle can be used as a clinical indicator of improved blood glucose control.

## Introduction

Diabetes is one of the fastest-growing global health challenges of the 21st century. Type 1 diabetes (T1DM) has become a significant global disease burden. According to the Global Burden of Disease study, in 2021, there were 19.6 million T1DM patients worldwide, with over 530,000 new cases. Among them, China had 1.44 million T1DM patients and over 32,000 new cases, ranking it third in the world (1).

The close relationship between progress and the development of microvascular, macrovascular, and neuropathic complications with glucose control has been extensively studied in patients with type 1 diabetes (2, 3). Data from the China T1DM Study indicates that patients with ketoacidosis still account for 41.1%, the rate of blood glucose control among hospitalized patients is only 12%, and the prevalence of chronic complications reaches 60.7% (4). Thus, exploring safer and more stable blood glucose management programs is worthwhile.

It has been demonstrated that numerous factors contribute to poor glycemic control, including older age, female sex, alcohol consumption, a higher body mass index (BMI), smoking, a longer duration of the disease, lower physical activity, failure to adhere to therapeutic recommendations, and numerous others (5). Hemoglobin A1c(HbA1c), a product of glucose binding with hemoglobin in red blood cells, reflects average blood sugar levels over the past two to three months. Its clinical significance includes evaluating blood sugar control, aiding in the diagnosis of diabetes, predicting risks of complications, guiding adjustments to treatment plans, and distinguishing between stress-induced hyperglycemia (6). Several factors influence HbA1c levels, including not only the average blood glucose level of patients but also the lifespan of red blood cells, age, race, and ethnicity. The HbA1c level can be impacted by the red blood cell (RBC) lifecycle. Since HbA1c is a result of the non-enzymatic chemical reaction between hemoglobin and blood glucose, alterations in hemoglobin can influence HbA1c levels. Research has indicated that RBC transfusions can lead to a decrease in HbA1c levels (7–9). The prevalence of dyslipidemia and diabetes mellitus among individuals over 30 years of age in Korea has continuously increased. A study found an association between high glycated hemoglobin levels and a diagnosis of dyslipidemia among Korean adults (10). Another analysis revealed the relationship between sedentary behavior, diastolic blood pressure, and HbA1c levels among a specific cohort of young female university students. In their study, sedentary hours and diastolic blood pressure were independently associated with HbA1c levels (11).

Bioelectrical impedance analysis (BIA) is a non-invasive method that assesses the electrical impedance properties of body tissues and has been widely applied in body composition evaluation. Among the parameters derived from BIA, Phase angle (PhA) is considered one of the most comprehensive indicators reflecting cellular health status. Phase angle is calculated from the phase shift between reactance and resistance, and its magnitude reflects the capacitive properties of cell membranes as well as the distribution of intracellular and extracellular water. From a biological perspective, Phase angle essentially represents an integrated manifestation of cell membrane integrity, cell quantity, and cellular functional status (12).

A higher Phase angle generally indicates intact cell membrane structure, a higher proportion of intracellular water, and favorable cellular metabolic activity. In contrast, a reduced Phase angle is commonly interpreted as a sign of impaired membrane permeability, decreased cellular function, or a relative increase in extracellular water. Therefore, Phase angle is not merely a body composition parameter but is increasingly regarded as a functional indicator reflecting cellular-level health. Previous studies have demonstrated that a lower Phase angle is closely associated with enhanced inflammatory responses, elevated oxidative stress, malnutrition, reduced muscle mass, and long-term metabolic disturbances, and it has been linked to adverse outcomes in various chronic diseases (13, 14).

Existing research has primarily focused on populations with type 2 diabetes mellitus (T2DM). Multiple studies have reported a significant negative association between Phase angle and glycated hemoglobin (HbA1c) levels, indicating that poorer glycaemic control is associated with lower phase angle values (15). In addition, Phase angle has been reported to be closely related to chronic diabetic complications such as neuropathy, nephropathy, and sarcopenia, suggesting that Phase angle reflects not only metabolic status but may also be associated with disease progression and tissue damage (16). These findings provide important evidence supporting the potential role of Phase angle as a biomarker in diabetes management.

However, compared with T2DM, evidence regarding Phase angle in type 1 diabetes mellitus (T1DM) remains markedly limited. T1DM is characterized by distinct pathophysiological features, primarily autoimmune-mediated destruction of pancreatic β-cells, earlier disease onset, and lifelong dependence on exogenous insulin therapy. Long-term glycaemic fluctuations, insulin dose adjustments, and sustained metabolic stress may exert cumulative effects on cellular structure and function. Therefore, investigating whether Phase angle can reflect glycaemic control in adults with T1DM and serve as a potential early-warning indicator is of significant clinical relevance.

## Materials and methods

### Trial design and patient enrollment

This cross-sectional study was conducted between September 2023 and May 2024. The subjects of the study were patients with T1DM in the Department of Endocrinology and Metabolism at the First People’s Hospital of Yunnan Province. The inclusion criteria were as follows: (1) Meeting the diagnostic criteria for T1DM as defined in the Expert Consensus on Adult T1DM Management (17); (2) Being fully conscious with normal communication skills and able to cooperate with study-related measurements and physical examinations; (3) An age range of 18–70 years; (4) No previous history of gastrointestinal diseases or digestive tract surgeries; (5) A disease duration of at least 1 year. The exclusion criteria included: (1) Inability to complete examinations; (2) Pregnant or breastfeeding women; (3) Long-term use of thyroid medications, steroids, or estrogen/progestogen drugs; (4) Implantation of cardiac pacemakers, cardioverter-defibrillators, or replacement joints.

### Procedures and measurements

Upon admission, comprehensive baseline data were systematically collected, including demographic characteristics (age, sex), anthropometric parameters (height, weight, waist circumference, hip circumference), and clinical information (diagnosis, disease duration).

### Body composition analysis and phase angle

We evaluated body composition and phase angle using a human body composition analyzer (Inbody S10), which employs the BIA method. Participants fasted for a minimum of 8 hours and emptied their bladders on the morning of the test. Measurement procedure: Clean the electrode contact surfaces of the instrument, measure the participant’s height and weight; barefoot, wipe the soles and palms of both hands with an alcohol swab, and ensure the feet and hands make maximum contact with the instrument’s silver electrode surfaces to introduce the electric current. After the measurement is completed, the phase angle value at a frequency of 50 kHz is obtained.

### Laboratory indicators include HbA1c levels

The analytical methods for the biochemical measurements were as follows: total cholesterol, HDL-c, triglycerides, and LDL-c were measured using the enzymatic colorimetric method; HbA1c was determined by high-performance liquid chromatography; total protein was assayed by the diacuprim method, albumin by the bromophenol green method, and prealbumin by the immunoturbidimetric method.

### Statistical analysis

All statistical analyses were performed using SPSS software (version 26.0; IBM Corp). Continuous variables with a normal distribution were presented as mean ± standard deviation ( $\bar{\text{x}}$ ± s) and compared using an independent samples t-test. Non-normally distributed continuous variables were expressed as median with interquartile range [M (P25, P75)]. Categorical variables were reported as frequencies and percentages (n,%).

Pearson’s and Spearman’s correlation coefficients were utilized to assess the relationship between the dependent variable and independent variables. Linear regression models, both crude and adjusted, were employed to ascertain the strength of the association between the dependent variable and independent variables. Model fit was evaluated using the coefficient of determination (R²), with values closer to 1 indicating a better fit to the observed data.

## Results

### Basic characteristics of patients

56 adults with T1DM were included in this study. **Table 1** summarizes the clinical data of the adults with T1DM. The ages of the men and women are 37.91 ± 15.13 years and 40.50 (21.00, 52.00) years, respectively, and their HbA1c levels are 10.05 ± 2.85% and 9.79 ± 1.94%, respectively. The phase angles are 6.00 (5.07, 6.80) for men and 5.16 ± 0.93 for women.

**Table 1:** Characteristics of T1DM.

Variables	Male(n=22)	Female(n=34)	P
Age (years)	37.91 ± 15.13	40.50(21.00,52.00)	0.840
Height (cm)	168.77 ± 6.56	158.00(155.75,162.00)	<0.001
Weight (kg)	60.31 ± 10.29	51.85 ± 7.24	<0.001
BMI (kg/m^2^)	21.23 ± 3.40	20.40 ± 2.75	0.320
Total protein (g/L)	64.05 ± 8.73	69.11(63.60,74.15)	0.067
Albumin (g/L)	39.70 ± 6.39	41.66 ± 5.19	0.213
Prealbumin (mg/L)	194.11 ± 43.74	189.93 ± 47.42	0.741
HbA1c (%)	10.05 ± 2.85	9.79 ± 1.94	0.668
Total cholesterol (mmol/L)	4.34 ± 0.98	4.99 ± 1.07	0.027
Triglyceride (mmol/L)	0.96(0.69,1.62)	0.86(0.66,1.41)	0.586
HDL-C (mmol/L)	1.22 ± 0.32	1.67 ± 0.52	<0.001
LDL-C (mmol/L)	2.55 ± 0.75	2.76 ± 0.84	0.362
Waistline (cm)	82.11 ± 9.57	76.30 ± 8.69	0.023
Hip(cm)	92.22 ± 7.67	89.48 ± 6.70	0.164
Waist-hip ratio(%)	88.93 ± 6.01	85.34 ± 7.79	0.072
Phase angle (°)	6.00(5.07,6.80)	5.16 ± 0.93	0.027

### Phase angle analysis results

Pearson’s or Spearman’s correlation coefficient analyses were used to measure the association between Phase angle and parameters, including HbA1c levels, as shown in **Table 2**. In males, a positive correlation was observed between the Phase angle and several indicators, including total protein, albumin, prealbumin, total cholesterol, and hip circumference, even after adjusting for age and weight. Conversely, the Phase angle and HbA1c levels showed a negative correlation, even after adjusting for age and weight, as indicated in **Table 3**. In females, a positive correlation was observed between the Phase angle and albumin, even after adjusting for age and weight. Similarly, the Phase angle and HbA1c levels exhibited a negative correlation, even after adjusting for age and weight.

**Table 2:** Pearson’s and partial correlation between phase angle and metabolic parameters (male).

Variables Phase angle (°)	Correlation coefficient		Partial correlation coefficient	
	r	p	r	p
Total protein (g/L)	0.509	0.016	0.611	0.004
Albumin (g/L)	0.613	0.002	0.641	0.002
Prealbumin (mg/L)	0.462	0.030	0.609	0.004
HbA1c (%)	-0.488	0.021	-0.552	0.012
Total cholesterol mmol/L)	0.500	0.018	0.703	<0.001
Triglyceride (mmol/L)	0.211	0.346	0.222	0.347
HDL-C (mmol/L)	0.368	0.092	0.608	0.004
LDL-C (mmol/L)	0.469	0.028	0.592	0.006
Waistline (cm)	0.510	0.015	0.177	0.456
Hip circumference(cm)	0.624	0.002	0.485	0.030
Waist-hip ratio(%)	0.117	0.603	-0.212	0.369

Partial correlation in control of age and weight.

Phase angle: Outcome variable.

HDL, high-density lipoprotein; LDL, low-density lipoprotein; HbA1c, glycated hemoglobin.

**Table 3:** Pearson’s and partial correlation between phase angle and metabolic parameters (female).

Variables Phase angle (°)	Correlation coefficient		Partial correlation coefficient	
	r	p	r	p
Total protein (g/L)	0.311	0.073	0.387	0.029
Albumin (g/L)	0.459	0.006	0.539	0.001
Prealbumin (mg/L)	0.160	0.365	0.251	0.166
HbA1c (%)	-0.374	0.029	-0.381	0.032
Total cholesterol mmol/L)	0.105	0.555	0.107	0.559
Triglyceride (mmol/L)	-0.144	0.416	-0.163	0.372
HDL-C (mmol/L)	0.246	0.160	0.259	0.152
LDL-C (mmol/L)	-0.029	0.873	-0.025	0.891
Waistline (cm)	0.008	0.962	-0.086	0.639
Hip circumference(cm)	0.167	0.344	0.150	0.413
Waist-hip ratio(%)	-0.126	0.476	-0.188	0.304

Partial correlation in control of age and weight.

Phase angle: Outcome variable.

HDL, high-density lipoprotein; LDL, low-density lipoprotein; HbA1c, glycated hemoglobin.

Simple linear regression and hierarchical regression analysis were used to measure the strength of the association between Phase angle and parameters, including HbA1c levels.

**Table 4** illustrates that in males, simple linear regression analyses revealed a positive association between Phase angle and several indicators, including total protein, albumin, prealbumin, total cholesterol, LDL-C, waist circumference, and hip circumference. After adjusting for age and weight using hierarchical regression analysis, several indicators maintained a significant positive association with Phase angle. Additionally, Phase angle and HbA1c levels initially showed a negative correlation; however, after adjusting for age and weight, the negative correlation persisted.

**Table 4:** Simple and adjusted linear regression (male).

Variables Phase angle (°)	Simple linear regression			Adjusted linear regression		
	*ß*	*r^2^*	*p*	*ß*	*r^2^*	*p*
Total protein (g/L)	0.070	0.259	0.016	0.073	0.523	0.004
Albumin (g/L)	0.115	0.375	0.002	0.016	0.052	0.002
Prealbumin (mg/L)	0.013	0.174	0.030	0.017	0.442	0.004
HbA1c (%)	-0.204	0.238	0.021	-0.482	0.384	0.012
Total cholesterol mmol/L)	0.605	0.250	0.018	0.769	0.551	<0.001
Triglyceride (mmol/L)	0.364	0.045	0.346	0.335	0.157	0.347
HDL-C (mmol/L)	1.336	0.135	0.092	2.007	0.441	0.004
LDL-C (mmol/L)	0.738	0.220	0.028	0.823	0.424	0.006
Waistline (cm)	0.064	0.260	0.015	0.052	0.141	0.456
Hip circumference(cm)	0.097	0.389	0.002	0.445	0.312	0.030
Waist-hip ratio(%)	2.334	0.014	0.603	-4.641	0.153	0.369

Adjusted with age and weight.

Phase angle: Outcome variable.

HDL, high-density lipoprotein; LDL, low-density lipoprotein; HbA1c, glycated hemoglobin.

**Table 5** indicates that in females, simple linear regression analyses revealed a positive association between Phase angle and albumin. Upon adjusting for age and weight using hierarchical regression analysis, the association between Phase angle and albumin remained significant and positive. Furthermore, Phase angle and HbA1c levels exhibited a negative correlation, which continued to be observed after adjusting for age and weight.

**Table 5:** Simple and adjusted linear regression (female).

Variables Phase angle (°)	Simple linear regression			Adjusted linear regression		
	*ß*	*r^2^*	*p*	*ß*	*r^2^*	*p*
Total protein (g/L)	0.023	0.097	0.073	0.031	0.162	0.004
Albumin (g/L)	0.082	0.211	0.006	0.104	0.301	0.002
Prealbumin (mg/L)	0.003	0.026	0.365	0.006	0.77	0.004
HbA1c (%)	-0.179	0.140	0.029	-0.187	0.158	0.012
Total cholesterol (mmol/L)	0.091	0.011	0.555	0.092	0.026	<0.001
Triglyceride (mmol/L)	-0.175	0.021	0.416	-0.020	0.041	0.347
HDL-C (mmol/L)	0.434	0.061	0.160	0.464	0.081	0.004
LDL-C (mmol/L)	-0.031	0.001	0.873	-0.028	0.015	0.006
Waistline (cm)	0.001	0.001	0.962	-0.012	0.022	0.456
Hip circumference(cm)	0.023	0.028	0.344	0.029	0.037	0.030
Waist-hip ratio(%)	-1.510	0.016	0.476	-2.413	0.049	0.369

Adjusted with age and weight.

Phase angle: Outcome variable.

HDL, high-density lipoprotein; LDL, low-density lipoprotein; HbA1c, glycated hemoglobin.

## Discussion

In this study, we aimed to explore the relationship between Phase angle and HbA1c levels, in adult T1DM. The findings of this study revealed a negative correlation between Phase angle and HbA1c in males, even after adjusting for age and weight. At the same time, this study also revealed a negative correlation between Phase angle and HbA1c in females. This implies that higher HbA1c levels are associated with lower Phase angle in adult T1DM. This relationship suggests that Phase angle can be used as a clinical observational indicator of improved blood glucose control.

As a core parameter of bioelectrical impedance analysis (BIA), Phase angle (PhA) is theoretically associated with the capacitive properties of cell membranes, the distribution of intracellular and extracellular water, and body cell mass, thereby reflecting cell membrane integrity and overall cellular function. From a clinical perspective, PhA tends to decrease in conditions characterized by reduced muscle mass or cell mass, increased inflammatory or metabolic stress, and abnormal fluid distribution (such as an increased extracellular water proportion) (13). Therefore, PhA can be regarded as a composite indicator of overall health status. Phase angle exhibits a decline in value under certain ill health conditions, including cancer, and viral infections et al (18). These diseases are associated with damage to the body’s cells, causing a decrease in the capacity of cells to capture electrical energy. Its clinical significance has been increasingly recognized, particularly in elderly, bedridden, and edematous patients, as well as in those experiencing declines in cognitive and physical function (19–21). In our study, a positive correlation was observed between the phase angle and several indicators in males, including total protein, albumin, prealbumin, total cholesterol, and hip circumference. A positive correlation was observed between the Phase angle and albumin in females. A population-based study conducted on adults showed that an increase in WC is likely to decrease the Phase angle value (22). But in our study, only in males was there a positive correlation between hip circumference and Phase angle. A study to evaluate the relationship between Phase angle and nutritional status and the prognostic significance of Phase angle in critically ill cancer patients showed that a positive correlation was ascertained between the phase angle and albumin (23). In a study to determine the association between Phase angle and nutritional status in community-dwelling patients with type 2 diabetes, concluding observations that Phase angle was positively associated with albumin, a nutritional assessment index (24).

In this study, Phase angle values were higher in males than in females. This finding is biologically plausible and may be related to sex-specific differences in body composition and cellular characteristics, which is consistent with previous reports (25). One possible explanation is that males generally have higher fat-free mass and skeletal muscle mass and a lower relative fat mass. Because phase angle is considered to reflect cell membrane integrity, cellular health, and the distribution of intracellular and extracellular water, greater muscle mass and higher intracellular water content in males may contribute to their higher phase angle values. In addition, differences in sex hormones, particularly their effects on muscle metabolism and body water distribution, may also play a role.

The main findings of this study are consistent with previous results reported in populations with type 2 diabetes mellitus (T2DM), in which several studies have demonstrated a negative association between Phase angle and HbA1c (26, 27). The present study extends this observation to individuals with type 1 diabetes mellitus (T1DM). T1DM and T2DM differ fundamentally in their pathophysiological mechanisms: T1DM is characterized by autoimmune destruction of pancreatic β-cells, requiring lifelong dependence on exogenous insulin and being associated with greater glycemic variability and a higher risk of hypoglycemia. In T1DM, changes in PhA may therefore be more strongly related to glycemic fluctuations, protein catabolism, alterations in lean body mass, and the burden of diabetes-related complications. Despite these differences, our findings indicate that the pattern of association between PhA and glycemic control is similar in both types of diabetes, suggesting that PhA may reflect a shared pathological basis of diabetic metabolic dysregulation—namely, the detrimental effects of chronic hyperglycemia on cellular structure and function (28). Moreover, this association has also been observed in pediatric populations (29) and in other special groups, such as young Japanese sumo wrestlers (30), further supporting the broad applicability of PhA as an important biomarker for assessing metabolic health across diverse populations.

This study has several limitations. First, although the main analyses were based on prespecified hypotheses, Bonferroni correction for multiple comparisons was not applied, which may increase the risk of type I error. Therefore, the results should be interpreted with caution, and future studies with larger sample sizes or independent populations are needed to further validate our findings. Second, due to the cross-sectional study design, although an association between phase angle and glycated hemoglobin levels was observed, a causal relationship cannot be established. Third, several key factors that may influence glycemic control and phase angle, such as diabetes duration, daily insulin dose, and educational level, were not included in the analysis. In addition, lifestyle-related factors, including physical activity status, exercise intensity, and nutritional intake, were not assessed, and these unmeasured variables may have introduced potential residual confounding.

Future studies should adopt longitudinal designs to more comprehensively elucidate the factors influencing phase angle and glycated hemoglobin levels, and to determine whether temporal changes in phase angle can predict subsequent deterioration in glycemic control. Moreover, it would be valuable to explore whether interventions such as nutritional modification and structured physical exercise can simultaneously improve glycemic variability and increase phase angle, thereby providing further evidence to support comprehensive management strategies for patients with type 1 diabetes.

## Conclusion

This study has, for the first time, demonstrated a negative correlation between Phase angle and HbA1C levels in patients with T1DM. Beyond being a simple body composition parameter, Phase angle serves as a biomarker reflecting cellular health, nutritional status, and metabolic stability. Incorporating Phase angle into comprehensive evaluation systems for patients with type 1 diabetes could provide clinicians with a novel perspective, enabling more precise and personalized glucose management and ultimately improving long-term patient outcomes.

## Data Availability

The original contributions presented in the study are included in the article/supplementary material. Further inquiries can be directed to the corresponding author.
